# SickleInAfrica Consortium: A Seven-Country Study Evaluating the Performance of Dried Blood Spot Point-of-Care Testing in Newborn Screening for Sickle Cell Disease

**DOI:** 10.1080/03630269.2026.2630709

**Published:** 2026-04-09

**Authors:** Obiageli Eunice Nnodu, Wilson Mupfururirwa, Sarah Kiguli, Lulu Chirande, Boubacar Ali Toure, Fred Stephen Sarfo, Patience Kuona, Aldiouma Guindo, Andrew Louden, Arthemon Nguweneza, Emmanuel Balandya, Catherine Chunda-Liyoka, Victoria Nembaware, Chandré Oosterwyk, Mario Jonas, Jack Morrice, Andre Pascal Kengne, Siana Nkya, Upend Masamu, Julie Makani, Patrick Ohiani Moru, Abdulmalik Koya, Heriud Martin, Lawson Chikara, Evans Xorse Amuzu, Natasha Kaweme, Mary Nakibirango, Chinwe Okeke, Maxwell Nwegbu, Yaa Oppong-Mensah, Deogratias Munube, Reuben Ikechukwu Chianumba, Anazoeze Madu, Ruth Namazzi, Gwendoline Kandawasvika, Nesla Mahenge, Bruno Mbando, Agnes Jonathan, Josephine Mgaya, Janeth Manongi, Irene Minja, Emmanuel Peprah, Ambroise Wonkam

**Affiliations:** a Department of Haematology and Blood Transfusion, University of Abuja & Centre of Excellence for Sickle Cell Disease Research and Training (CESRTA), University of Abuja, Abuja, Nigeria; b SickleInAfrica Data Coordinating Centre (SADaCC), University of Cape Town, Cape Town, South Africa; c Department of Paediatrics and Child Health, Makerere University Faculty of Medicine, Kampala, Uganda; d Muhimbili University of Health and Allied Sciences, Dar es Salaam, Tanzania; e Centre de Recherche et Lutte contre la Drépanocytose (CRLD), Bamako, Mali; f Kwame Nkrumah University of Science and Technology, Kumasi, Ghana; g Faculty of Medicine and Health Sciences, University of Zimbabwe, Harare, Zimbabwe; h National Heart, Lung, and Blood Institute, Bethesda, Maryland, USA; i University of Zambia-School of Medicine, Lusaka, Zambia; j School of Medicine, Yaounde, Cameroon; k Department of Haematology and Immunology, College of Medicine, University of Nigeria, Enugu, Nigeria; l Christoffel-Blindenmission(CBM) International, Tanzania; m School of Global Public Health, New York University, New York, New York, USA

**Keywords:** Sickle cell disease, newborn screening, dried blood spot, point-of-care testing, SickleInAfrica

## Abstract

Sickle cell disease (SCD) remains a major public health concern in sub-Saharan Africa (SSA), where approximately 200,000 newborns are affected annually. Without early diagnosis and access to care, up to 50% of these children may die before the age of five. Although newborn screening (NBS) programs have proven effective in improving survival, their implementation across Africa is constrained by logistical barriers associated with standard diagnostic methods such as isoelectric focusing (IEF), high-performance liquid chromatography (HPLC), and cellulose acetate electrophoresis. Dried blood spot point-of-care testing (DBS-POCT) offers a potentially scalable alternative due to its stability, simplicity, and suitability for centralized analysis. We evaluated the diagnostic accuracy of DBS-POCT using the HemoTypeSC test compared to both standard POCT and reference laboratory testing across 705 newborns (0–3 months old) in seven countries within the SickleInAfrica Consortium. DBS-POCT demonstrated high sensitivity and specificity for detecting HbAA and HbAS, moderate sensitivity for HbSS, and lower sensitivity for HbAC, with some variability across countries. In several countries, DBS-POCT outperformed standard POCT, particularly in detecting SCD subtypes. Our findings support the utility of DBS-POCT for expanding newborn screening programs in resource-limited settings.

## Introduction

Sickle cell disease (SCD) is caused by an inherited single-nucleotide substitution in both copies of the β-globin gene [*HBB*(Glu7Val)]. The sickle-cell mutation results in the production of an abnormal β-globin chain of hemoglobin. The latter accounts for approximately 95% of red blood cell (RBC) dry weight and serves as the primary oxygen-carrying molecule in the blood [1]. Sickle hemoglobin (HbS) tends to polymerize, deforming RBCs into rigid, sickle-shaped cells that obstruct small blood vessels, causing hypoxia in critical organs. Sickled RBCs also break down easily, reducing their lifespan from the standard 120 days to an average of 20 days. These two events, vaso-occlusion and hemolysis, cause individuals with SCD to suffer recurrent pain, chronic anemia, and multiple organ damage, leading to significant morbidity and early mortality. As a result of its partial protection against *Plasmodium falciparum* malaria, *HBB*(Glu7Val) has become prevalent in malaria-endemic regions worldwide. Annually, nearly 300,000 newborns are diagnosed with SCD worldwide, with 75% of these cases occurring in Africa. Despite high under-five mortality rates in Africa (30%–50%) and a stagnant life expectancy (up to the 5th decade), among adult patients in the USA, the number of individuals with SCD globally has risen by 41.4% over the past 20 years, reaching 7.74 million in 2021. Consequently, there is an increasing burden and a significant research gap [1-4].

Newborn screening (NBS) for SCD and early interventions can reduce mortality in children under five years of age [5]. In SSA, pilot newborn screening projects have been conducted in several countries, but have not been adopted by governments for national rollout. Consequently, most African countries, except Egypt, do not have a national newborn screening (NBS) program. The next opportunity in Africa lies in building capacity for implementing research on the scalability of evidence-based interventions, including NBS, penicillin prophylaxis, the adoption of hydroxyurea therapy, pain management, blood transfusions, transcranial Doppler screening, and patient and caregiver education for SCD management [6,7]. High disease prevalence in Africa positions this region to lead epidemiological, translational, and clinical research studies on SCD.

The diagnostic methods for SCD include cellulose acetate electrophoresis (CAE), isoelectric focusing (IEF), capillary electrophoresis (CE), high-performance liquid chromatography (HPLC), tandem mass spectrometry, and molecular studies (Frommel 2018) [8]. Apart from CAE, the equipment and reagents for these tests are expensive, require stable electricity, a high level of training for both performance and interpretation, and centralization. These pose barriers to scaling up across all levels of care in the African setting and to uptake by governments [9-11].

Point-of-care tests (POCTs) are medical diagnostic tests performed at or near the site where a patient receives care. These tests provide immediate results, allowing for rapid decision-making and treatment adjustments. POCTs are designed to be easy to use, require minimal training, and often do not need specialized laboratory equipment. POCTs have recently been developed to overcome barriers of conventional screening programs [12,13]. A study in Nigeria demonstrated that screening with a point-of-care test (POCT) is feasible and acceptable to both healthcare providers and parents [7]. However, in busy immunization clinics, two or three personnel are typically involved in the process, making it more costly and potentially reducing the effective use of standard POCT techniques in mass screening.

POCT using Dried Blood Spots (DBS-POCT) combines the convenience of point-of-care testing (POCT) with the stability and transportability of dried blood spots. The process involves collecting blood via heel stick or finger prick, spotting it onto filter paper, drying it, and then either testing it onsite or transporting it to the hospital laboratory for batch testing. DBS-POCT is a cheap, minimally invasive blood sampling technique used in standard NBS programs [14,15]. Another study in Nigeria explored the use of DBS-POCT with POCT to achieve throughput and found 100% concordance between standard POCT and DBS-POCT for HbAA, HbAS, HbAC, and HbSC identification. DBS-POCT has been extensively and successfully used in Africa as a tool for the collection and transportation of blood specimens for the diagnosis of HIV. Finding a reliable POCT for SCD that uses DBS-POCT will potentially expand testing for SCD to include samples collected in other public health programs like the Prevention of Maternal to Child Transmission of HIV, thus integrating NBS for SCD into that program.

The sensitivity and specificity of POCTs have been evaluated in several countries [15,16,17,18,19,20] (Table 1). HemoTypeSC is relatively cheaper than other available POCTs, though it has some challenges, such as reverse interpretation.

This multicenter study is aimed at validating a DBS-POCT (HemoTypeSC) for newborn screening (0–28 days) and early infant diagnosis (29 days–3 months) for broader scale-up in SSA and identifying the primary barriers and facilitators to the implementation of SCD NBS programs at primary healthcare facilities across seven SickleInAfrica countries (Nigeria, Mali, Tanzania, Ghana, Uganda, Zimbabwe, and Zambia), using POCT applied to DBS-POCT.

This paper is based on the preliminary phase where the performance characteristics of DBS-POCT were tested against standard HemoTypeSC and IEF/HPLC on 100 newborns in each country, making a total of 700 in all. Following this, the quantitative phase using DBS-POCT HemoTypeSC further tested 900 newborns across participating countries, while Nigeria tested 2,500 newborns.

## Materials and methods

### Study design

This was an exploratory sequential mixed-methods assessment of the use of three technologies (Standard HemoTypeSC POCTs, DBS-POCT, and IEF/HPLC) to facilitate newborn screening for SCD.

### Study population

The study population comprised newborns aged 0–3 months from all SickleInAfrica Consortium sites in Ghana, Nigeria, Tanzania, Mali, Uganda, Zimbabwe, and Zambia.

### Screening sites

Screening for participants took place in the immunization clinics, labor and postnatal wards.

### Inclusion criteria

All newborns aged 0–3 months attending immunization clinics or labor and postnatal wards, with parents/guardians who provided informed consent, were eligible to participate in the study.

### Exclusion criteria

Unwell babies at risk of bleeding from the puncture sites were excluded from the study.

### Sample size

The initial phase involved approximately 100 samples taken from newborns and infants in each participating country to evaluate the performance characteristics of DBS-POCT.

The methodology for this study has recently been published (10.1136/bmjopen-2024-089056). Ethical clearance was obtained from **NIGERIA-** NHREC/01/01/2007–13/02/2024B (National Health Research Ethics Committee of Nigeria), UGANDA-Mak-SOMREC-2022–526 (Makerere University School of Medicine Ethical Review Committee, Uganda) **Uganda**, N°2021/106/CE/USTTB (Ethics Committee of University of Sciences Techniques and Technologies of Bamako) **Mali**, CHRPE/AP/033/21 (Committee on Human Research, Publication and Ethics, Kumasi) **Ghana**, MUHAS-REC-05–2023-1686 (Muhimbili University Of Health And Allied Sciences Research and Ethics Committee, Dar Es Salaam) **Tanzania**, MOH/UTH/CH/TJ(Ministry of Health) Republic of **Zambia**, Parirenyatwa Group of Hospitals, **Zimbabwe**.

### Procedures

The initial step involved using HemoTypeSC to screen for SCD on-site (in the immunization clinic and post-natal ward) using heel-prick blood, and the results were communicated to participants on the same day. Screening with HemoTypeSC was conducted (by nurses, midwives, and research assistants) following the manufacturer’s guide [16]. Subsequently, DBS-POCT samples were collected from newborns and infants at the postnatal ward and immunization clinics, air-dried for a minimum of 3 h at 18 °C–25 °C, and shipped to a clinical laboratory for testing.

**Figure 1.**
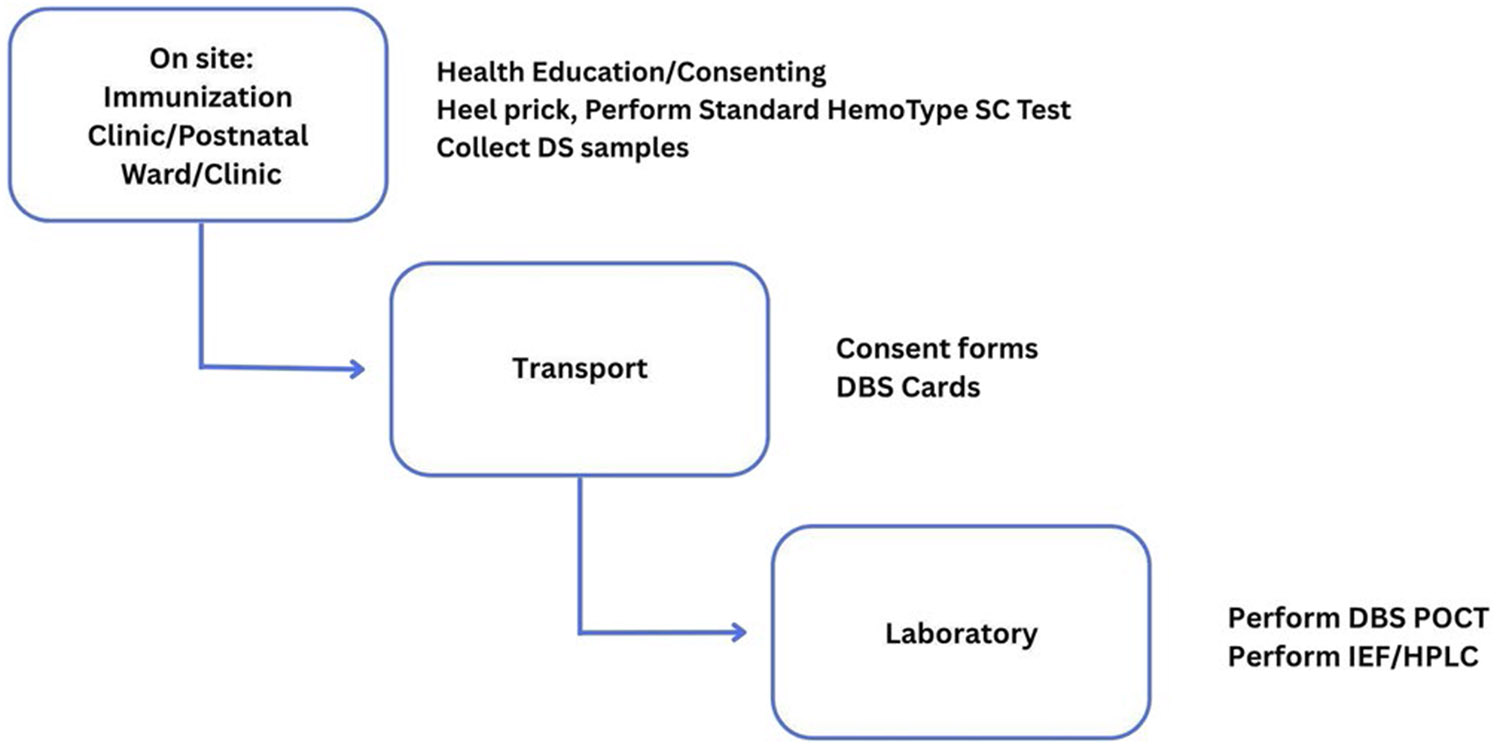
Workflow for newborn and early infant screening using DBS-POCT and standard reference testing. The diagram illustrates the procedural flow from on-site recruitment (e.g. immunization clinics or postnatal wards), where health education, consenting, heel prick sampling, and standard HemoTypeSC testing are conducted. DBS samples and consent forms are then transported to the laboratory for further analysis using DBS-based POCT and confirmatory reference tests (IEF or HPLC).

The DBS was eluted, and the HemoTypeSC^™^ standard protocol was followed to determine SCD status [15]. DBS-POCT-identified SCD-positive samples were confirmed by Isoelectric Focusing (IEF) or High-Performance Liquid Chromatography (HPLC), following the manufacturer’s guide [15]. Analysis comparing Standard POCT/DBS-POCT with IEF/HPLC was then performed (Figure 1).

### Statistical analysis

This study employs two diagnostic methods—Standard HemoTypeSC POCT and DBS-POCT—to screen newborns for SCD. A Reference Standard (RS), specifically IEF/HPLC, is used as a validation benchmark to evaluate the accuracy and reliability of these screening methods. Both HemoTypeSC POCT and DBS-POCT are independently compared with IEF/HPLC. Additionally, although each participating country functioned as a distinct study site, Zimbabwe and Zambia operate as a single joint site within the SickleInAfrica consortium, sharing the same implementation team, training structure, and data management system. Consequently, their results were analyzed together for all comparisons.

## Results

### Study enrollment and population

A total of 705 newborns aged 0 to 3 months were enrolled across the seven SickleInAfrica countries: Nigeria, Ghana, Mali, Tanzania, Uganda, Zimbabwe, and Zambia. Participant recruitment was carried out in immunization clinics, labor wards, and postnatal wards at all sites. Each country enrolled approximately 100 newborns during the preliminary phase. The sex distribution was balanced overall, with 51.3% male and 48.7% female (Table 2).

### SCD subtypes

Reference testing using IEF or HPLC revealed that the majority of newborns (80.6%) exhibited the HbAA subtype. Heterozygous HbAS was present in 12.6% of cases, while HbSS and HbAC accounted for 1.3% and 3.0%, respectively. The remaining results were from other genotypes and indeterminate results (2.6%). Country-level variation in the subtype frequencies was observed, with some sites reporting higher proportions of HbAS or HbAC, reflecting regional differences in subtype distribution (Table 3).

### Overall diagnostic performance of DBS-POCT and POCT

**Figure 2.**
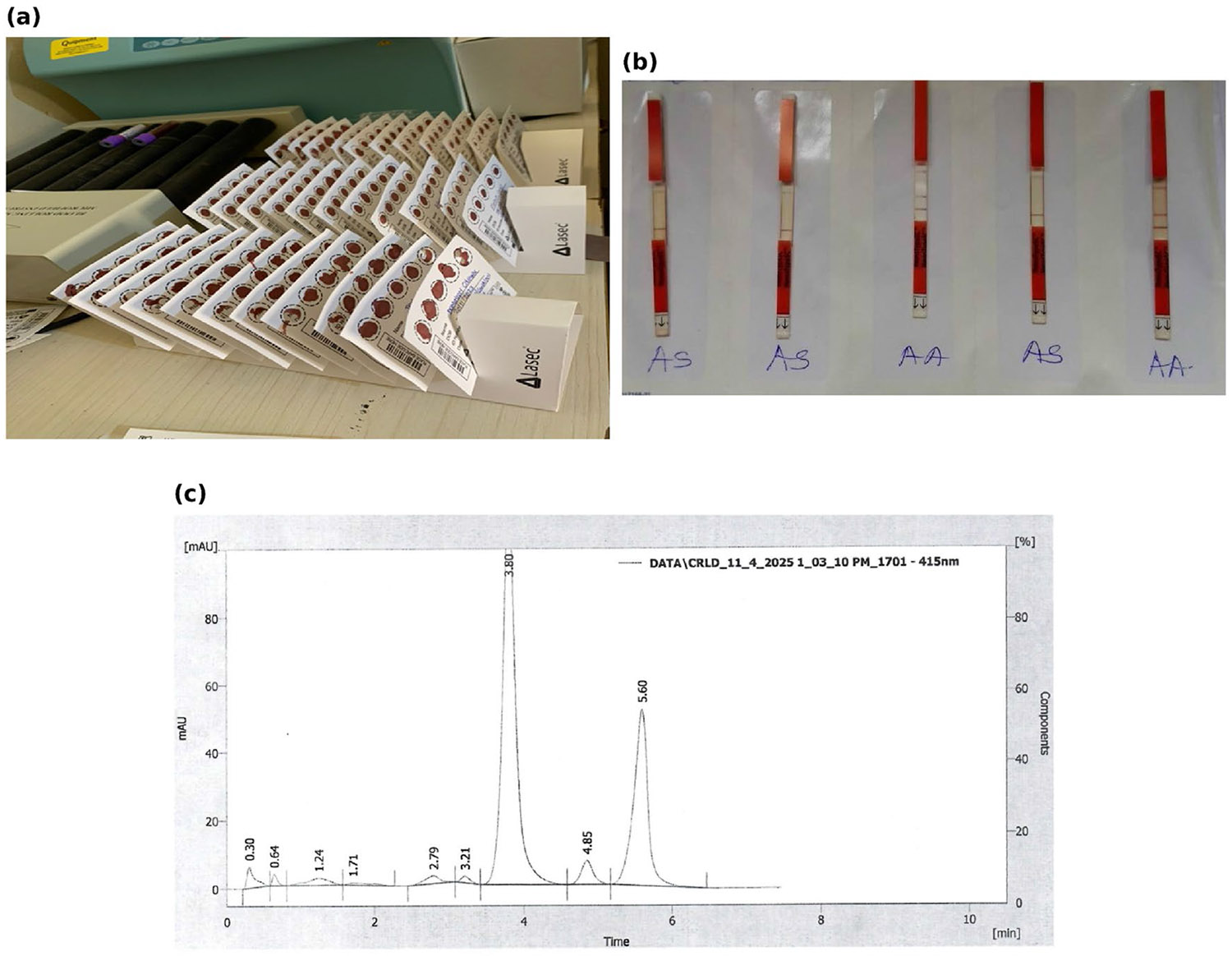
Visual overview of sample collection materials and hemoglobin testing procedures. (a) Dried blood spot (DBS) cards following sample collection and drying prior to laboratory processing. (b) Representative HemoTypeSC rapid diagnostic test strips illustrating typical screening results for newborn samples. (c) Example high-performance liquid chromatography (HPLC) chromatogram showing distinct HbA and HbS peaks consistent with the AS sickle cell trait subtype.

The diagnostic accuracy of both DBS-POCT and standard POCT was evaluated by comparison with the reference standard. At the consortium level, DBS-POCT demonstrated high sensitivity for HbAA (0.95; 95% CI: 0.93–0.97) and HbAS (0.93; 95% CI: 0.86–0.98), moderate sensitivity for HbSS (0.78; 95% CI: 0.40–0.97), and low sensitivity for HbAC (0.39; 95% CI: 0.17–0.64). Specificity was high across all subtypes, with values of 0.89 for HbAA, 1.00 for HbSS, 0.94 for HbAS, and 1.00 for HbAC. Positive predictive values (PPV) were also high, particularly for HbAA (0.98), HbSS (0.88), and HbAC (0.88), while PPV for HbAS was slightly lower at 0.72. Negative predictive values (NPV) were strong across all subtypes, reaching 1.00 for HbSS and ≥0.93 for the others (Figure 2).

In contrast, the conventional POCT displayed very high sensitivity for HbAA (0.98; 95% CI: 0.97–0.99) but had reduced sensitivity for HbAS (0.85; 95% CI: 0.76–0.92), HbSS (0.50; 95% CI: 0.19–0.81), and HbAC (0.50; 95% CI: 0.25–0.75). Specificity remained high across all subtypes (≥0.83), and the NPV values were similarly strong, exceeding 0.90 for all subtypes. However, PPV for HbSS was noticeably lower at 0.71, indicating a greater chance of false positives (Table 4).

### Country-level performance of DBS-POCT and POCT

In Nigeria, both DBS-POCT and POCT demonstrated perfect diagnostic performance for HbAA, and the SCD subtypes HbAS, and HbSS, with sensitivity, specificity, PPV, and NPV values all equal to or approaching 1.00. For HbAA, both methods achieved sensitivity and specificity of 1.00 and 0.92, respectively, with a PPV of 0.97 and an NPV of 1.00. HbAS and HbSS were also detected with perfect accuracy. Neither test detected any HbAC cases, and the sensitivity for AC was 0.00 for both DBS-POCT and POCT, though specificity and NPV for AC remained high at 1.00 and 0.98, respectively.

In Tanzania, DBS-POCT outperformed standard POCT across nearly all SCD subtypes. DBS-POCT demonstrated perfect or near-perfect sensitivity for HbAA (0.99), HbAS (1.00), and HbSS (1.00), with corresponding specificity values of 1.00, 0.99, and 1.00. The PPVs for HbAA, HbAS, and HbSS were 1.00, 0.94, and 1.00, respectively, and NPVs ranged from 0.94 to 1.00. By contrast, POCT showed reduced sensitivity for AS (0.67) and failed to detect any SS cases, resulting in an SS sensitivity of 0.00. Despite this, POCT specificity remained high for all subtypes (≥0.94), and NPV for SS was 0.98.

In Uganda, DBS-POCT maintained a strong overall performance. For HbAA, DBS-POCT achieved a sensitivity of 0.96 and specificity of 0.96, with a PPV of 0.99 and NPV of 0.88. Sensitivity for HbAS was 0.95, with a specificity of 0.96, PPV of 0.86, and NPV of 0.99. DBS-POCT also detected HbSS with 100% sensitivity and specificity, with both PPV and NPV equal to 1.00. POCT had reduced sensitivity for HbSS (0.67), though its specificity remained high (0.99), with a PPV of 0.67 and NPV of 0.99. For HbAS, POCT yielded a sensitivity of 0.80, specificity of 0.95, and corresponding PPV and NPV values of 0.80 and 0.95, respectively.

In Mali, DBS-POCT showed high sensitivity for HbAA (1.00), HbAS (0.80), and HbAC (0.88), with specificity values of 0.92, 1.00, and 0.99, respectively. The PPV for AA was 0.99, while the NPV was 1.00. For AC, PPV and NPV were 0.88 and 0.99, respectively. POCT performance was comparable for HbAA and HbAS, though it showed reduced sensitivity for AC (0.67), with a PPV of 1.00 and NPV of 0.99. Both methods maintained high specificity across SCD subtypes.

In Ghana, both tests showed limited diagnostic performance, particularly for less common SCD subtypes. DBS-POCT failed to detect any SS or AC cases, with SS and AC sensitivities of 0.00. Sensitivity for HbAA was 0.67, with a specificity of 0.65, PPV of 0.86, and NPV of 0.38. HbAS was detected with 0.86 sensitivity and 0.67 specificity; however, the PPV was low at 0.31, despite a high NPV of 0.96. For POCT, SS detection sensitivity was modest at 0.50, while AC detection improved slightly to 0.55. The PPVs for SS and AC were 1.00 and 1.00, respectively, while their NPVs were 0.99 and 0.95. HbAA and HbAS were identified by POCT with sensitivities of 0.96 and 0.86, respectively.

In Zimbabwe and Zambia, DBS-POCT demonstrated high sensitivity for HbAA (0.99), HbAS (0.86), and HbAC (0.98), with specificity exceeding 0.93 across all SCD subtypes. The PPV for AA and AC was 0.99 or higher, while NPV values remained above 0.93. DBS-POCT failed to detect the single HbSS case present at this site, resulting in a sensitivity of 0.00 for SS, though specificity and NPV remained perfect. POCT demonstrated similar performance patterns, missing the HbSS case entirely. Nevertheless, POCT achieved strong sensitivity and specificity for AA (0.99 and 0.87, respectively) and AS (0.86 and 0.99), with PPV and NPV values all exceeding 0.93 (Supplementary Table S1).

### Test concordance and statistical comparison between DBS-POCT and POCT

To assess overall concordance between DBS-POCT and POCT, the SCD subtypes were grouped as SCD (HbSS, HbAS) or non-SCD (HbAA, HbAC, HbCC, indeterminate, or other). Although the two tests consistently classified most newborns, a number of discordant cases were observed. DBS-POCT identified more newborns as having SCD than POCT. Statistical comparison using McNemar’s Chi-Square test indicated a significant difference in classification between the two methods (χ^2^ = 14.38, df = 1, *p* < 0.001), with an odds ratio of 3.7 (95% CI: 1.84–7.44), suggesting that DBS-POCT was significantly more likely than POCT to detect SCD and may offer improved sensitivity for screening in this setting.

### Training of healthcare workers

Healthcare workers varied across the participating countries (Table 5). In Nigeria, the trained healthcare workers included medical doctors, nurses, and laboratory technicians. In Tanzania, nurses received the training under the supervision of a medical doctor. In Mali, laboratory technicians carried out the study. In Uganda and Ghana, healthcare workers, including nurses, laboratory technicians, and data collectors, received training and conducted the procedure. In Zimbabwe and Zambia, medical doctors were trained through a combination of videos and in-person practice. All the key personnel traveled to Abuja for training on the study protocol, and the DBS-POCT before the commencement of the study using standardized training materials based on the manufacturer’s official HemoTypeSC instructional video and printed resources. However, detailed information on the prior experience, knowledge, or level of supervision or evaluation of the trained healthcare workers was not systematically recorded in all the countries. The variation observed likely reflects a combination of operator experience, local supervision, and contextual differences across implementation settings.

## Discussion

This study aimed to evaluate the diagnostic performance of different NBS methods for SCD across diverse public health settings in seven SickleInAfrica consortium countries: Ghana, Mali, Nigeria, Tanzania, Uganda, Zambia, and Zimbabwe. A comparative analysis was conducted to assess the diagnostic accuracy of POCT using HemoTypeSC and DBS-POCT, using IEF or HPLC as the reference standard. By implementing these methods in routine care settings such as immunization clinics, labor wards, and postnatal units, the study provides practical insight into the feasibility and performance of each diagnostic approach in resource-limited settings.

The findings revealed that DBS-POCT demonstrated strong diagnostic performance overall and, in several settings, outperformed standard POCT, particularly in detecting SCD. While both methods showed high specificity and negative predictive value, DBS-POCT identified more cases of HbSS and HbAS compared to POCT. This difference was statistically significant, as confirmed by McNemar’s test, with DBS-POCT being significantly more likely to detect affected individuals. Such increased sensitivity is critical in the context of NBS, where missed diagnoses may delay the initiation of lifesaving interventions, such as penicillin prophylaxis, parental education, and enrollment in comprehensive care. The higher sensitivity of DBS-POCT in this study aligns with findings from other evaluations of HemoTypeSC and similar platforms, including studies in Nigeria [16], Mali [17], and the Democratic Republic of Congo [18], which reported high accuracy but also highlighted variability depending on site conditions and test implementation protocols.

The analysis of overall concordance between DBS-POCT and standard POCT revealed a statistically significant difference in classification performance. Although the two methods agreed in the majority of cases, DBS-POCT was more likely to classify newborns as having SCD. McNemar’s Chi-Square test indicated that newborns identified by DBS-POCT were over three times more likely to be missed by standard POCT. While overall agreement between the methods was high, this discordance highlights the superior sensitivity of DBS-POCT. It reinforces the risk of underdiagnosis when relying solely on standard POCT in clinical practice.

Country-level analysis revealed notable variation in diagnostic accuracy: DBS-POCT maintained high sensitivity and specificity across most sites, whereas POCT alone showed lower sensitivity in several settings, particularly for HbSS. In Tanzania and Uganda, for instance, POCT failed to detect several confirmed SS cases, whereas DBS-POCT correctly identified them. These discrepancies underscore the importance of considering not only the intrinsic test characteristics but also the operational environment, including user training, lighting, humidity, storage conditions, and sample handling - all of which can impact the real-world performance of rapid diagnostics. Similar implementation challenges have been noted in pilot NBS programs in SSA, where variability in test performance often reflects gaps in infrastructure or human resource capacity [6,12].

Differences in test accuracy across sites might also be associated with the category of healthcare personnel performing the tests and the training they receive. DBS-POCT was typically performed by laboratory technicians with formal training and greater experience in diagnostic workflows, while standard POCT was often conducted by nurses or midwives in postnatal wards and immunization clinics. These staff members may have had less familiarity with test interpretation, particularly for assays such as HemoTypeSC, which use reverse interpretation principles. Sites such as Nigeria, Mali, and Uganda achieved higher diagnostic accuracy, likely reflecting differences in how training was implemented locally rather than differences in training content. Although all countries used the same standardized study protocol, the same manufacturer-produced HemoTypeSC training materials, and participated in a central hands-on training session before implementation, the extent of on-site supervision, hands-on practice, and overall accuracy varied between individuals and thus countries. Since operator-level details such as professional background, prior experience, and competency assessment were not systematically recorded, the study cannot determine the precise reasons for inter-site discrepancies in POCT performance.

However, all countries used the same standardized study protocol, training materials, and all participated in a central hands-on training session in Nigeria before implementation; the extent of further hands-on practice, supervision, and refresher exposure at individual sites was not documented. Thus, in this preliminary phase, it was not possible to assess how variation in experience or training intensity may have influenced diagnostic accuracy.

Although IEF and HPLC are recognized gold standards for newborn screening, they are not entirely error-free. IEF is sensitive to pre-analytical and procedural factors such as sample quality, staining, washing, and manual interpretation, while HPLC may yield misclassifications when variant peaks co-migrate, have similar retention times (RTs) and elute in the same ‘window’ on the chromatogram. Since our population consisted of newborns under three months of age, elevated HbF levels were expected. HemoTypeSC is blind to HbF as it targets only HbA, HbS, and HbC antigens. It reduces the resolution of low-abundance adult hemoglobin fractions on IEF/HPLC. The small number of abnormal hemoglobin subtypes detected may also have amplified apparent error rates. Consequently, some discrepancies between DBS-POCT/POCT and the reference standard may reflect limitations of the reference assays rather than POCT performance.

Another limitation of this study is the absence of molecular confirmation by a centralized reference laboratory, despite the collection of dried blood spots. Genetic testing methods, such as PCR-based assays or targeted sequencing of the *HBB* gene, provide the highest level of diagnostic precision and could have served as an additional gold standard for verifying the hemoglobin SCD subtypes. However, the primary objective of this implementation-focused, international collaborative study was to evaluate the diagnostic performance under routine conditions in sub-Saharan Africa. Capacity for molecular studies is limited, and IEF or HPLC remains the standard confirmatory test. Future studies incorporating centralized genetic validation would strengthen quality assurance frameworks and further refine the diagnostic accuracy.

The consistently high specificity and NPV of both DBS-POCT and POCT suggest that these tools are reliable in ruling out SCD when the result is negative. However, the reduced sensitivity of standard POCT in some settings raises concerns for its use as a standalone screening method. False negatives in newborn screening can have significant consequences, particularly in resource-constrained settings where missed cases may not be identified until severe complications occur. In contrast, DBS-POCT may offer a more robust alternative by leveraging the stability of DBS, reducing sample degradation during transport, and enabling centralized retesting or confirmatory analysis where necessary. This is especially relevant in countries lacking on-site laboratory capacity, where integration with other DBS-based programs (e.g.early infant HIV diagnosis) may offer logistical advantages [14,15].

The performance of DBS-POCT compared to standard POCT likely reflects a combination of operational and environmental factors rather than differences in assay chemistry. DBS-POCT samples were analyzed in controlled laboratory environments by trained personnel, reducing errors related to lighting, humidity, and user interpretation that can affect bedside testing. In contrast, standard POCT was conducted in busy clinical areas such as postnatal wards and immunization clinics, where conditions and user expertise varied widely. While DBS-POCT involves delayed testing and longer turnaround times, it offers advantages in quality assurance, batch processing, and central oversight, particularly when integrated into existing DBS transport networks used for early infant HIV diagnosis or metabolic screening. However, this approach also introduces delays in dispatching results to families and slightly higher per-test costs, which are offset by simplified supply chains that require only the test kit rather than multiple reagents. Conversely, immediate POCT enables same-day parental counseling and referral but may require longer, competency-based training and formal certification to maintain diagnostic accuracy across diverse care settings.

Importantly, our findings support the potential of DBS-POCT as a practical and scalable option for expanding newborn screening for SCD in SSA. Unlike standard POCTs, which require on-site interpretation and are prone to user-related variability, DBS samples can be collected in remote clinics and transported to centralized laboratories for batch processing, facilitating broader coverage - particularly in rural or resource-constrained settings. Given its superior performance across several countries, DBS-POCT offers a reliable approach for early detection of SCD. When integrated into existing maternal and child health services, it could significantly improve early diagnosis and care for affected newborns [11].

## Conclusions

Our findings confirm that DBS-POCT shows high sensitivity for the HbAA and HbAS subtypes, moderate sensitivity for HbSS, and lower sensitivity for HbAC. Specificity remained consistently high across all SCD subtypes, with strong negative predictive values, indicating that negative DBS results are highly reliable. Notably, DBS-POCT was more effective than standard POCT in detecting individuals with HbAA, HbAS, and HbSS in most participating countries.

Performance varied by subtype and implementation context. While pooled results showed reduced sensitivity for HbAC, sites with more experience and better-trained staff demonstrated stronger diagnostic accuracy across all detected subtypes. This underscores the importance of proper training and skilled personnel to ensure optimal use of the test in routine public health settings. Together, the findings support DBS-POCT as a reliable and scalable diagnostic tool for expanding newborn screening programs for SCD in SSA.

## Supplementary Material

Supp 1

Supplemental data for this article can be accessed online at https://doi.org/10.1080/03630269.2026.2630709.

## Figures and Tables

**Table 1.** Comparative sensitivity and specificity of POCT methods across other studies, evaluated against gold-standard laboratory testing (IEF, HPLC, or PCR).

Study	Setting/population	Method compared	SCD subtypes evaluated	Sensitivity (%)	Specificity (%)
Okeke *et al.*, 2022	Nigeria	DBS-POCT vs PCR	AA, AS, SS, AC	100%	100%
Nnodu *et al.*, 2019	Nigeria	Fresh blood POCT vs electrophoresis	AA, AS, SS	93.4%	99.9%
Guindo *et al.*, 2024	Mali	Cord blood POCT vs HPLC	AA, AS, SS, HbC, HbS/β+	81.67%	99.69%
Steele *et al.*, 2019	Nigeria, DRC, India	Fresh blood POCT (HemoTypeSC) vs IEF	AA, AS, SS	100%	100%
Olatunya *et al.*, 2021	Nigeria	Fresh blood POCT vs PCR	AA, AS, SS	100%	100%
Segbena *et al.*, 2018	Mali and Togo	SickleSCAN^®^ POCT vs HPLC	AA, AS, SS	97%–100%	98%–100%

**Table 2.** The average age and sex distribution of the babies screened across the consortium ranged from day-old to 1.4 months, with noticeable differences between the various sites.

	Nigeria	Tanzania	Mali	Uganda	Ghana	Zim/ Zam
Total number of samples	100	100	104	100	101	200
Average age in months(intervals)	1.4 (0.2–2.1)	0.0 (0–0)	0.2 (0.2–0.2)	0.7 (0.5–0.9)	1.0 (1.0–1.0)	–
Male (%)	51 (51)	50 (50)	48 (46.2)	55 (55.6)	45 (45)	105 (53.5)
Female (%)	49 (49)	50 (50)	56 (53.8)	44 (44.4)	55 (55)	93 (46.5)

The sex of the babies was balanced with slight variations in the percentages of male (45% to 55.6%) and female babies (44%–55%) across the countries.

**Table 3.** Summary of SCD subtype frequencies by country and diagnostic method (Reference Standard, POCT, and DBS-POCT).

	Nigeria (IEF)	Tanzania (IEF)	Mali (HPLC)	Uganda (IEF)	Ghana (IEF)	Zim/ Zam (IEF)	Total
Total	100	100	104	100	101	200	705
SCD subtype count—reference standard(RF)							
AA	74	81	86	71	71	185	568
SS	2	2	0	4	0	1	9
AS	22	14	5	20	14	14	89
AC	2	0	8	0	11	0	21
SCD subtype count (POCT)							
AA	76	83	86	76	74	186	581
SS	2	1	0	3	1	0	7
AS	22	14	5	19	17	14	91
AC	0	1	3	0	6	0	10
SCD subtype count (DBS-POCT)							
AA	76	80	87	74	55	185	557
SS	2	2	0	3	0	1	8
AS	22	15	5	21	39	14	116
AC	0	0	9	0	0	0	9

**Table 4.** Diagnostic performance of DBS-POCT and POCT methods across all study sites.

SCD subtype	Sensitivity [95% CI]	Specificity [95% CI]	PPV [95% CI]	NPV [95% CI]
DBS-POCT				
AA	0.95 [0.93–0.97]	0.89 [0.82–0.94]	0.98 [0.96–0.99]	0.78 [0.70–0.85]
SS	0.78 [040 – 0.97]	1.00 [0.99–1.00]	0.88 [0.47–1.00]	1.00 [0.99–1.00]
AS	0.93 [0.86–0.98]	0.94 [0.92–0.96]	0.72 [0.63–0.80]	0.99 [0.98–1.00]
AC	0.39 [0.17–0.64]	1.00 [0.99–1.00]	0.88 [0.47–1.00]	0.98 [0.97–0.99]
POCT				
AA	0.98 [0.97–0.99]	0.83 [0.75–0.90]	0.97 [0.95–0.98]	0.90 [0.82–0.95]
SS	0.50 [0.19–0.81]	1.00 [0.99–1.00]	0.71 [0.29–0.96]	0.99 [0.98–1.00]
AS	0.85 [0.76–0.92]	0.97 [0.96–0.99]	0.84 [0.74–0.90]	0.98 [0.96–0.99]
AC	0.50 [0.25–0.75]	1.00 [0.99–1.00]	0.89 [0.52–1.00]	0.99 [0.98–0.99]

Sensitivity, specificity, positive predictive value (PPV), and negative predictive value (NPV) are reported with 95% confidence intervals (CI) for each SCD subtype (AA, SS, AS, and AC).

**Table 5.** Summary of healthcare worker categories and training modalities across countries participating in the study.

Country	Training format	Categories of healthcare workers
Nigeria	Video tutorials + in-person demonstration	Medical doctors, nurses, laboratory technicians
Tanzania	Video tutorials + in-person demonstration	Nurses
Mali	Video tutorials + in-person demonstration	Laboratory technicians
Uganda	Video tutorials + in-person demonstration	Nurses, laboratory technicians, data collectors
Ghana	Video tutorials + in-person demonstration	Nurses, laboratory technicians, data collectors
Zim/Zam	Video tutorials + in-person demonstration	Medical doctors

## Data Availability

The data that support the findings of this study are available from the corresponding author, ON, upon reasonable request.
