# Supplementary material for: SickleInAfrica Consortium: A Seven-Country Study Evaluating the Performance of Dried Blood Spot Point-of-Care Testing in Newborn Screening for Sickle Cell Disease
Source: Hemoglobin. Author manuscript; Available in PMC 2026 Apr 22. (PMC13101935; doi:10.1080/03630269.2026.2630709)
Supplement: Supp 1 [file NIHMS2161658-supplement-Supp_1.docx]

| **Country** | **Genotype** | **Test** | **Sensitivity** | **Specificity** | **PPV** | **NPV** | **Test** | **Sensitivity** | **Specificity** | **PPV** | **NPV** |
| --- | --- | --- | --- | --- | --- | --- | --- | --- | --- | --- | --- |
| Nigeria | AA | DBS | 1.00  [0.95 - 1.00] | 0.92  [0.75 - 0.99] | 0.97  [0.91 - 1.00] | 1.00  [0.86 - 1.00] | POCT | 1.00  [0.95 - 1.00] | 0.92  [0.75 - 0.99] | 0.97  [0.91 - 1.00] | 1.00  [0.86 - 1.00] |
|  | SS |  | 1.00  [0.16 - 1.00] | 1.00  [0.96 - 1.00] | 1.00  [0.16 - 1.00] | 1.00  [0.96 - 1.00] |  | 1.00  [0.16 - 1.00] | 1.00  [0.96 - 1.00] | 1.00  [0.16 - 1.00] | 1.00  [0.96 - 1.00] |
|  | AS |  | 1.00  [0.85 - 1.00] | 1.00  [0.95 - 1.00] | 1.00  [0.85 - 1.00] | 1.00  [0.95 - 1.00] |  | 1.00  [0.85 - 1.00] | 1.00  [0.95 - 1.00] | 1.00  [0.85 - 1.00] | 1.00  [0.95 - 1.00] |
|  | AC |  | 0.00  [0.00 - 0.84] | 1.00  [0.96 - 1.00] | - | 0.98  [0.93 - 1.00] |  | 0.00  [0.00 - 0.84] | 1.00  [0.96 - 1.00] | - | 0.98  [0.93 - 1.00] |
| Ghana | AA |  | 0.67  [0.55 - 0.78] | 0.65  [0.43 - 0.84] | 0.86  [0.74 - 0.94] | 0.38  [0.23 - 0.55] |  | 0.96  [0.88 - 0.99] | 0.78  [058 - 0.91] | 0.92  [0.83 - 0.97] | 0.88  [0.68 - 0.97] |
|  | SS |  | 0.00  [0.00 - 0.98] | 1.00  [0.96 - 1.00] | - | 0.98  [0.94 - 1.00] |  | 0.50  [0.01 - 0.99] | 1.00  [0.96 - 1.00] | 1.00  [0.03 - 1.00] | 0.99  [0.94 - 1.00] |
|  | AS |  | 0.86  [0.57 - 0.98] | 0.67  [0.56 - 0.77] | 0.31  [0.17 - 0.48] | 0.96  [0.88 - 1.00] |  | 0.86  [0.57 - 0.98] | 0.94  [0.87 - 0.98] | 0.71  [0.44 - 0.90] | 0.98  [0.91 - 1.00] |
|  | AC |  | 0.00  [0.00 - 0.37] | 1.00  [0.96 - 1.00] | - | 0.92  [0.84 - 0.96] |  | 0.55  [0.23 - 0.83] | 1.00  [0.96 - 1.00] | 1.00  [0.54 - 1.00] | 0.95  [0.88 - 0.98] |
| Tanzania | AA |  | 0.99  [0.93 - 1.00] | 1.00  [0.80 - 1.00] | 1.00  [0.96 - 1.00] | 0.94  [0.73 - 1.00] |  | 0.95  [0.88 - 0.99] | 0.71  [0.44 - 0.90] | 0.94  [0.86 - 0.98] | 0.75  [0.48 - 0.93] |
|  | SS |  | 1.00  [0.16 - 1.00] | 1.00  [0.96 - 1.00] | 1.00  [0.16 - 1.00] | 1.00  [0.96 - 1.00] |  | 0.00  [0.00 - 0.84] | 0.99  [0.94 - 1.00] | 0.00  [0.00 - 0.98] | 0.98  [0.93 - 1.00] |
|  | AS |  | 1.00  [0.78 - 1.00] | 0.99  [0.94 - 1.00] | 0.94  [0.70 - 1.00] | 1.00  [0.96 - 1.00] |  | 0.67  [0.38 - 0.88] | 0.95  [0.88 - 0.99] | 0.71  [0.42 - 0.92] | 0.94  [0.87 - 0.98] |
|  | AC |  | - | 1.00  [0.96 - 1.00] | - | 1.00  [0.96 - 1.00] |  | - | 0.99  [0.94 - 1.00] | 0.00  [0.00 - 0.98] | 1.00  [0.96 - 1.00] |
| Uganda | AA |  | 0.96  [0.88 - 0.99] | 0.96  [0.78 - 1.00] | 0.99  [0.92 - 1.00] | 0.88  [0.69 - 0.97] |  | 0.96  [0.88 - 0.99] | 0.87  [0.66 - 0.97] | 0.96  [0.88 - 0.99] | 0.87  [0.66 - 0.97] |
|  | SS |  | 1.00  [0.29 - 1.00] | 1.00  [0.96 - 1.00] | 1.00  [0.29 - 1.00] | 1.00  [0.96 - 1.00] |  | 0.67  [0.09 - 0.99] | 0.99  [0.94 - 1.00] | 0.67  [0.09 - 0.99] | 0.99  [0.94 - 1.00] |
|  | AS |  | 0.95  [0.75 - 1.00] | 0.96  [0.89 - 0.99] | 0.86  [0.65 - 0.97] | 0.99  [0.93 - 1.00] |  | 0.80  [0.56 - 0.94] | 0.95  [0.87 - 0.99] | 0.80  [0.56 - 0.94] | 0.95  [0.87 - 0.99] |
|  | AC |  | - | 1.00  [0.96 - 1.00] | - | 1.00  [0.96 - 1.00] |  | - | 1.00  [0.96 - 1.00] | - | 1.00  [0.96 - 1.00] |
| Mali | AA |  | 1.00  [0.96 - 1.00] | 0.92  [0.64 - 1.00] | 0.99  [0.94 - 1.00] | 1.00  [0.74 - 1.00] |  | 1.00  [0.96 - 1.00] | 0.86  [0.42 - 1.00] | 0.99  [0.94 - 1.00] | 1.00  [0.54 - 1.00] |
|  | SS |  | - | 1.00  [0.96 - 1.00] | - | 1.00  [0.96 - 1.00] |  | - | 1.00  [0.96 - 1.00] | - | 1.00  [0.96 - 1.00] |
|  | AS |  | 0.80  [0.28 - 0.99] | 1.00  [0.96 - 1.00] | 1.00  [0.40 - 1.00] | 0.99  [0.94 - 1.00] |  | 1.00  [0.40 - 1.00] | 1.00  [0.96 - 1.00] | 1.00  [0.40 - 1.00] | 1.00  [0.96 - 1.00] |
|  | AC |  | 0.88  [0.47 - 1.00] | 0.99  [0.94 - 1.00] | 0.88  [0.47 - 1.00] | 0.99  [0.94 - 1.00] |  | 0.67  [0.09 - 0.99] | 1.00  [0.96 - 1.00] | 1.00  [0.16 - 1.00] | 0.99  [0.94 - 1.00] |
| Zimbabwe/Zambia | AA |  | 0.99  [0.97 - 1.00] | 0.93  [0.68 - 1.00] | 0.99  [0.97 - 1.00] | 0.93  [0.68 - 1.00] |  | 0.99  [0.97 - 1.00] | 0.87  [0.60 - 0.98] | 0.99  [0.96 - 1.00] | 0.93  [0.66 - 1.00] |
|  | SS |  | 0.00 [0.00 - 0.98] | 0.99  [0.97 - 1.00] | 0.00  [0.00 - 0.98] | 0.99  [0.97 - 1.00] |  | 0.00  [0.00 - 0.98] | 1.00  [0.98 - 1.00] | - | 1.00  [0.97 - 1.00] |
|  | AS |  | 0.86  [0.57 - 0.98] | 0.99  [0.96 - 1.00] | 0.86  [0.57 - 0.98] | 0.99  [0.96 - 1.00] |  | 0.86  [0.57 - 0.98] | 0.99  [0.96 - 1.00] | 0.86  [0.57 - 0.98] | 0.99  [0.96 - 1.00] |
|  | AC |  | 0.98  [0.95 - 0.99] | 1.00  [0.98 - 1.00] | - | 1.00  [0.98 - 1.00] |  | - | 1.00  [0.98 - 1.00] | - | 1.00  [0.98 - 1.00] |

**Supplementary Table S1.** Diagnostic performance of DBS-POCT and POCT methods across countries and genotypes. Sensitivity, specificity, PPV, and NPV are presented with 95% confidence intervals (CIs) for each genotype (AA, AS, SS, AC) by test type.
